# Catastrophic health care spending and impoverishment in Kenya

**DOI:** 10.1186/1472-6963-12-413

**Published:** 2012-11-21

**Authors:** Jane Chuma, Thomas Maina

**Affiliations:** 1 Kenya Medical Research Institute (KEMRI)-Wellcome Trust Research Programme, Nairobi, Kenya; 2 Centre for Tropical Medicine, Nuffield Department of Clinical Medicine, University of Oxford, Oxford, United Kingdom; 3 Ministry of Medical Services, Nairobi, Kenya

## Abstract

**Background:**

Many health systems in Africa are funded primarily through out-of-pocket payments. Out-of-pocket payments prevent people from seeking care, can result to catastrophic health spending and lead to impoverishment. This paper estimates the burden of out-of-pocket payments in Kenya; the incidence and intensity of catastrophic health care expenditure and the effect of health spending on national poverty estimates.

**Methods:**

Data were drawn from a nationally representative health expenditure and utilization survey (n = 8414) conducted in 2007. The survey provided detailed information on out-of-pocket payments and consumption expenditure. Standard data analytical techniques were applied to estimate the incidence and intensity of catastrophic health expenditure. Various thresholds were applied to demonstrate the sensitivity of catastrophic measures.

**Results:**

Each year, Kenyan households spend over a tenth of their budget on health care payments. The burden of out-of-pocket payments is highest among the poor. The poorest households spent a third of their resources on health care payments each year compared to only 8% spent by the richest households. About 1.48 million Kenyans are pushed below the national poverty line due to health care payments.

**Conclusions:**

Kenyans are becoming poorer due to health care payments. The need to protect individuals from health care related impoverishment calls for urgent reforms in the Kenyan health system. An important policy question remains what health system reforms are needed in Kenya to ensure that financial risk protection for all is achieved.

## Background

Protecting households from catastrophic health care costs is a desirable objective of health systems worldwide. The World Health Organization (WHO) call for universal health coverage emphasized the need to protect households from catastrophic medical expenses and impoverishment arising from seeking health care [1]. The call also urged health systems to ensure that health care costs do not prevent people from receiving needed health services [2]. Globally it estimated that 150 million people suffer financial catastrophe each year due to health care payments and about 100 million are pushed into poverty because of out-of-pocket (OOP) payments [3].

Catastrophic health care payments occur in both rich and poor countries, but over 90% of the people affected reside in low-income countries [4]. Catastrophic health expenditure can occur regardless of the amount of money paid to health care services. Rich households might pay large medical bills without experiencing negative implications, while low levels of spending among poor households can have severe financial implications for livelihoods [4,5]. There is no single accepted definition of catastrophic spending. Some studies assess payments in relation to the budget share [6-8]; while others argue that catastrophic spending should be measured in relation to capacity to pay (i.e. household expenditure net of food spending) [4,9,10]. Nonetheless, all measures suggest that when households spend a large proportion of their budget on health care, they often forego other goods and services, which can have negative implications for living standards [11].

Health systems in Africa and other low-income countries are predominantly funded through OOP payments. Out-of-pocket payments do not offer any financial risk protection; many households incur high health expenditure, while others are impoverished due to health care costs [4]. A significant number of households do not seek care because they cannot afford to pay [12]. Households often adopt coping strategies to meet the costs associated with seeking care [13]. These strategies, although useful in the short-term, lead to impoverishment or deepen poverty among households who are already poor [5,6,14]. Such households are hardly captured in national poverty estimates, since high health costs raise their expenditure above the poverty threshold and are therefore considered to be non-poor [15].

Various studies assess the impact of catastrophic spending on household poverty. These studies, mainly conducted in Asia and Latin America, show that health care costs are major causes of impoverishment. In their study on health expenditures in 11 Asian countries, van Doorslaer et al. reported that poverty estimates were 14% higher when OOP payments are accounted for and that about 78 million people are pushed into poverty due to heath care costs [15]. Elsewhere, a survey of 89 countries found that catastrophic expenditure was reported by 3%, 1.8% and 0.6% of households in low, middle and high income countries respectively [3]. Few studies have documented the levels of catastrophic health expenditures in Africa. In Burkina Faso, about 15% of households reporting illness incurred costs greater than 40% of their non-food consumption expenditure [16], while in Uganda, 2.9% of households incurred catastrophic expenditure in 2003 [10]. In Nigeria, 40.2% of households incurred costs greater than 10% of their consumption expenditure; this proportion reduced to 14.8% when the threshold was set at 40% [17]. The poorest households were more likely to incur catastrophic expenditures compared to households.

A limitation of the few studies conducted in Africa is that they do not assess the implications of health care costs on national poverty estimates [10,16-18]. Assessing the role of health care payments on poverty is important for informing policy on the need to incorporate health financing designs in poverty reduction programmes and for highlighting the urgent need to ensure that health financing systems offer financial risk protection. This paper contributes to the literature by assessing the extent of catastrophic health spending and impoverishment in Kenya. Using different thresholds, which have been widely applied in the literature, the paper estimates the incidence and intensity of catastrophic health care expenditure for both outpatient and inpatient care and shows the proportion of individuals pushed into poverty due to OOP payments. Also estimated is the amount by which households resources would fall short of poverty thresholds.

## Methods

### Study setting

Kenya’s Gross Domestic Product (GDP) per capita was 739 US Dollars in 2009. The proportion of Kenyans living below the poverty line was 45.9% in 2005 [19], although recent statistics suggests that these levels could have increased to over 50% [20]. A description of the Kenyan health financing and delivery system is published elsewhere [21]. Briefly, all public health facilities charge user fees at the point of care. In 2004, user fees at dispensaries and health centres were replaced with a flat consultation fee of Kenya shillings 10 (US$0.13) and 20 (US$0.26) respectively. There exists a significant private sector that owns about 49% of all health facilities in the country. The Kenyan health system relies heavily on OOP payments, accounting for 51.1% of total health expenditure in 2001; 39.3% in 2005 and 36.7% in 2009 [22,23].

### Data sources

Data are from a nationally representative cross-sectional household survey conducted by the Ministry of Health in 2007. Detailed data were collected on socio-demographic characteristics, self reported illnesses, health care utilization patterns, OOP payments, sources of funds and consumption expenditure for both food and non-food items. Out-of-pocket spending were collected for various items including registration, drugs, consultation, diagnostic tests, surgery, daily bed rates among others. Data were collected for both outpatient and inpatient illnesses using a four week and one year recall period respectively.

### Data analysis

#### Measuring incidence and intensity of catastrophic spending

Standard approaches to assess the incidence and intensity of catastrophic expenditure and the implications for poverty estimates are described in detail by O’Donnell et al. ([2008]). These analytical approaches are briefly described here but interested readers are encouraged to refer to O’Donnell et al. ([2008]) for additional information.

Briefly, estimating catastrophic expenditure requires measuring the extent to which health costs exceed different thresholds of household income or consumption expenditure. The incidence of catastrophic spending can therefore be estimated from a fraction of a sample with health care costs as a share of total (or non-food) expenditure exceeding a certain threshold. There is no single accepted threshold for catastrophic health care payments. Often, the choice of the threshold is arbitrary but two commonly used ones are 10% of total income or 40% of non-food income (referred to as capacity to pay). Alternative catastrophic thresholds are presented in this study to demonstrate sensitivity of different measures. Analysis is done purely on out-of-pocket payments (i.e. payments made directly to providers), although it is recognized that indirect costs affect households significantly [5]. Where costs were fully covered through health insurance, the same were excluded in the analysis unless co-payments were made, in which case the co-payment was considered as OOP payments. Households were classified into socioeconomic quintiles using per capita consumption expenditure.

The incidence of catastrophic payments is defined as payments in excess of a threshold budget share. The catastrophic head count (HC) refers to the percentage of households incurring catastrophic payments and is estimated as follows [11]:

(1) $$HC=\frac{1}{N}\sum_{i=1}^{N}E$$

Where N is the sample size: E is an indicator equal to 1 if OOP payments of a household *i* as a proportion of its consumption expenditure (total or non-food) is greater than the threshold and zero otherwise. The HC estimates the proportion of households that have OOP payments above the threshold but does not measure the amount by which these payments exceed the chosen threshold. The catastrophic payment overshoot is estimated to give an indication of how much OOP payments exceed the threshold. The overshoot (O) is estimated as follows [11]:

(2) $$O_{i}=E_{i}\left(T_{i}/X_{i}-z\right)$$

Where T_*i*_ is the OOP payments of household *i*, X_*i *_is the household consumption expenditure (food or non-food) and z is the threshold budget share. Following this estimation, the average over shoot is [11]:

(3) $$O=\frac{1}{N}\sum_{i=1}^{N}O$$

The intensity of catastrophic expenditure is measured by the payment in excess of the threshold, averaged over all households exceeding that threshold. This measure, referred to as the mean positive overshoot (MPO) is equal to:

(4) $$MPO=\frac{O}{HC}$$

#### Adjusting catastrophic spending estimates for socio-economic status

A limitation of the head count and overshoot discussed in the previous section is that they do not differentiate between poor and rich households [11]. The headcount (*HC*) for example counts all households whose levels of OOP payments exceed a certain threshold equally. The overshoot (*O*) counts the payments in excess of the threshold equally, irrespective of whether these payments are made by poor or rich households [11]. High levels of OOP payments among rich households can be met through reducing spending on non-basic items like entertainment, while for poor households, even low levels of spending might require foregoing basic needs like food and education. Clearly, the opportunity costs of catastrophic health care payments will differ between rich and poor households.

To account for differences in the distribution of catastrophic payments between rich and poor households, results are presented for weighted and un-weighted head counts and overshoot. The distribution of catastrophic payments in relation to household welfare is measured by the concentration indices for *E_i_* (*C_E_*) and *O_i_* (*C_o_*). The concentration index ranges from −1 to +1. It is negative (positive) if the variable of interest is concentrated among the poor (rich) [24]. For example, a positive value of *C_E _*indicates a greater tendency for the richer households to exceed the payment threshold, while a negative value indicates that the poor are more likely to exceed the threshold. The distribution of the head count and overshoot can be adjusted for socio-economic differences by putting into consideration the concentration indices *C_E_* and *C_O _*[25]. The weighted head count (*HC^w^*) and overshoot (*O^w^*) measures are computed as:

$$HC^{w}=HC\left(1-C_{E}\right);O^{w}O\left(1-CO\right)\text{.}$$

Where normative interpretation of catastrophic payments are necessary, it is considered appropriate to give more weight to excess payments made by poorer households [26]. The weighted head count (*HC*^*w*^) gives a weight of two to the lowest consumption expenditure (income) and the weight declines linearly with the rank in socio-economic measure such that the richest households receive a weight of zero [11].

#### Health care spending and impoverishment

National and international poverty estimates usually do not take into account OOP payments for health care. The implication of OOP payments on poverty estimates are estimated by calculating poverty levels using consumption expenditure before making health care payments (i.e. gross of OOP payments) and after paying for health care (i.e. net OOP payments) [7]. Three measures are presented: (1) Poverty head count, which refers to the proportion of households living below the poverty line; (2) Poverty gap, referring to the aggregate of all shortfalls from the poverty line (i.e. the poverty head count multiplied by the average deficit of the poor from the poverty line); (3) Normalised poverty gap, which is obtained by dividing the poverty gap by the poverty line. The normalized poverty gap is useful for international comparisons across countries with different poverty lines and currency units [11]. These measurements require setting a poverty line and assessing the extent to which health care payments push households below the poverty line. The national poverty line of Kenya shillings (KES) 1257 per person per month was used to estimate poverty levels before and after health care payments. This poverty line has been criticized as being too low to meet the high costs of living [20], but remains the official rate used by the Kenyan government to estimate poverty levels and thus provides a good basis for comparisons.

Data were analyzed using STATA (Version 11.2) and ADEPT (version 4.1). Ethical clearance was sought from the Kenya Medical Research Institute (protocol number 1609).

## Results

### Out-of-pocket payments on outpatient and inpatient services

A total of 8,414 households took part in the cross-sectional survey. Illnesses in the four weeks preceding the survey were reported by 52.9% of households (Table 1). Hospital admissions were reported by 9.3% of households. About 3% of illnesses reported in the four weeks preceding the survey were not treated and 125 individuals (11.5%) requiring hospital admission were not admitted due to cash shortages. The concentration indices (CI) show that the richest households were more likely to report illnesses (CI = 0.01 for outpatient illnesses; 0.098 for inpatient), however these differences were only significant for inpatient care (p < 0.001). Differences between rural and urban areas were also only significant for hospital admissions (p < 0.001).

**Table 1:** Proportion of survey households reporting illness

	**Outpatient (n = 4449)**	**Inpatient (n = 784)**
Quintiles		
1	840 (18.9)	120 (15.3)
2	871 (19.6)	128 (16.3)
3	936 (21.0)	156 (19.9)
4	927 (20.8)	196 (25.0)
5	875 (19.7)	184 (23.5)
Concentration Index	0.01	0.098
Urban	1376 (30.9)	296 (37.8)
Rural	3073 (69.1)	488 (62.2)

Mean annual spending for all households regardless of whether they reported illness was KES 3526.7 for outpatient services and KES 8195.5 for hospital admissions (Table 2). Rich households spent significantly more money on health care than the poor (P < 0.001). For example, the poorest households spent a mean of KES 2217 on outpatient services, while the richest households spent KES 5345.9 (P < 0.001). Mean spending on outpatient services was significantly higher in urban than in rural areas (Table 1).

**Table 2:** Mean annual spending in Kenya shillings

	**Outpatient**		**Inpatient**		**Total**	
	**All households**	**Household reporting illness in last 4 weeks**	**All households**	**Household reporting hospitalization**	**All households**	**Household reporting illness**
Quintile						
1	2217.0	3587.6	991.8	8388.8	5354.9	7005.3
2	2298.4	3961.6	879.6	10470.2	3566.6	6364.2
3	3802.8	5863.4	947.4	6962.4	5556.3	7902.8
4	3971.1	5863.2	1528.4	11630.1	7296.0	10403.6
5	5345.9	11226.8	1981.3	15798.9	19209.1	30603.1
Concentration Index	0.179	0.229	0.171	0.13	0.316	0.329
Urban	5290.3	9625.9	1520.6	12326.5	16113.2	27414.8
Rural	2959.4	5483.9	1183.6	11211.4	5648.6	9355.8
All	3526.7	6506.0	1265.6	11516.0	8195.5	13813.0

**Figure 1:**
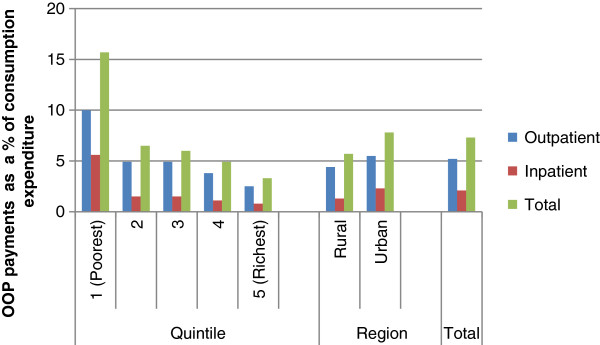
Mean household budget share of out of pocket payments.

Figure 1 shows the household budget share of OOP payments among households reporting illness. Overall households spent 5.2% of their annual budget on outpatient services and 2.0% on inpatient services. Mean total OOP payments amounted to 7.3% of households’ annual budget. The poorest households spent the largest share of their budget on health care (15.3%) compared to the richest households (3.3%). For outpatient services, the poorest quintile spent 10.0% of their budget on outpatient care, while the richest spent 2.5%. Hospital admissions consumed a lower share of household budget compared to outpatient care, with the poorest quintile spending about 5.6% of their budget on inpatient care, while the richest quintile spent 0.8%. Rural households spent a larger proportion of their annual budgets on health care compared to urban households (7.8% and 5.7% respectively).

### Catastrophic health expenditure and impoverishment

The incidence and intensity of catastrophic health payments are shown on Table 3. Results show an inverse relationship between catastrophic headcount and the various thresholds. For example, 15.5% of households reported total OOP payments exceeding 10% of total household expenditure. Increasing the threshold to 40% reduces the catastrophic head count to 4.6%. The incidence of catastrophic health expenditure increases when catastrophic payments are defined in respect to non-food budget. For instance, the proportion of household’s incurring total OOP payments greater than 25% increases from 6.9% to 16.0% when estimates are based on total and non-food expenditure respectively. The results also show that a larger proportion of households reporting illness in the four weeks preceding the survey (outpatient) incurred catastrophic expenditure compared to inpatient. For example, 4.4% of households reporting outpatient illnesses incurred costs above 25% of their total expenditure compared to 2.0% of households who reported a hospitalization.

**Table 3:** Incidence and intensity of catastrophic health expenditure

	**% of total expenditure**								
	**Outpatient**			**Inpatient**			**Total OOP payments**		
	**10%**	**25%**	**40%**	**10%**	**25%**	**40%**	**10%**	**25%**	**40%**
**Head count (HC)**									
1 (Poorest)	18.1	8.2	6.1	6.9	4.4	3.6	23.0	11.3	8.7
2	10.8	4.3	2.4	4.2	2.2	2.1	15.6	7.1	4.5
3	10.7	4.6	2.7	2.6	1.2	0.6	12.9	6.3	4.0
4	9.2	2.5	1.4	4.3	1.3	0.8	14.2	4.7	3.1
5 (Richest)	7.5	2.4	0.9	3.1	0.7	0.4	11.9	4.9	2.9
Total	11.2	4.4	2.7	4.2	2.0	1.5	15.5	6.9	4.6
**Weighted total head count**	13.3	5.6	3.7	4.9	2.7	2.2	17.6	8.3	5.8
**Overshoot**									
1 (Poorest)	20.0	18.4	17.5	3.5	2.9	2.5	34.5	32.3	31.0
2	3.0	1.9	1.5	1.8	1.4	1.0	6.2	4.6	3.8
3	2.9	1.9	1.4	0.6	0.3	0.2	4.7	3.3	2.6
4	1.5	0.8	0.5	0.7	0.4	0.2	3.9	2.6	2.0
5 (Richest)	1.2	0.5	0.3	0.4	0.1	0.0	6.2	5.0	4.5
Total	5.7	4.7	4.2	1.4	1.0	0.7	11.0	9.5	8.7
**Weighted overshoot (O**^**w**^**)**	9.5	8.2	7.6	2.06	1.59	1.35	15.0	13.3	12.4
**Concentration Index, C_E**	−0.184	−0.273	−0.382	−0.156	−0.385	−0.482	−0.137	−0.205	−0.257
**Concentration Index, C_O**	−0.663	−0.765	−0.817	−0.467	−0.580	−0.642	−0.489	−0.543	−0.574
**% of non food expenditure**									
	**Outpatient**			**Inpatient**			**Total OOP payments**		
	10%	25%	40%	10%	25%	40%	10%	25%	40%
**Head count**									
1 (Poorest)	35.6	22.3	17.0	10.9	9.7	9.3	39.7	26.6	21.1
2	23.0	13.9	9.4	7.6	5.6	4.1	28.3	18.1	12.8
3	22.1	11.5	8.5	4.5	3.3	2.5	24.8	14.2	10.7
4	16.7	7.9	4.9	7.2	3.5	1.9	22.6	12.6	7.7
5 (Richest)	12.0	4.8	2.5	4.8	2.4	0.9	17.9	8.4	4.8
Total	21.9	12.1	8.5	7.0	4.9	3.8	26.7	16.0	11.4
**Weighted total head count**	26.6	15.7	11.5	8.2	6.4	5.4	31.0	19.7	14.8
**Overshoot**									
1 (Poorest)	37.6	33.8	31.4	12.9	12.0	12.0	89.9	85.5	82.4
2	11.8	9.3	7.7	7.1	7.1	6.3	21.6	18.4	16.2
3	13.8	11.5	10.1	2.3	2.3	1.8	20.7	18.0	16.2
4	5.8	4.2	1.3	2.3	2.3	1.6	10.7	8.2	6.8
5 (Richest)	2.8	1.7	1.2	0.9	0.9	0.5	10.4	8.6	7.6
Total	14.2	11.9	10.6	5.0	5.0	4.4	30.1	27.2	25.4
**Weighted overshoot (O**^**w**^**)**	21.1	18.3	16.5	7.5	7.2	6.2	42.0	38.6	36.3
**Concentration index, C_E**	−0.214	−0.300	−0.352	−0.163	−0.311	−0.451	−0.162	−0.233	−0.293
**Concentration Index, C_O**	−0.487	−0.535	−0.567	−0.495	−0.552	0.585	−0.491	−0.525	−0.546

The weighted head count is higher than the un-weighted meaning that those who exceed the payment threshold tend to be poorer (Table 3). For example, the proportion of households with total OOP payments above 10% of their total expenditure is 15.5%. This proportion increases to 17.6% after applying weights to different socio-economic groups. Similarly, the proportion of households reporting total OOP payments above 40% of non-food spending is 11.4%. Accounting for differences in socio-economic status through the weighted head count increases this proportion to 14.8%. The weighted overshoot level presents a similar pattern for all threshold levels (i.e. they are higher than the un-weighted). The higher concentration of catastrophic payments among the poorest households is also confirmed by the negative values of concentration indices for the incidence of catastrophic payment (C_E). The C_E increases with the threshold, suggesting greater inequalities in catastrophic spending between the poorest and richest households for higher thresholds. The mean positive overshoot (i.e. the extent to which household health payments exceed various thresholds) show that on average, health expenditure for all households is 8.7% higher than 40% of total budget share. The corresponding value for non-food budget share is 25.4%.

The poverty head count before and after accounting for OOP payments is shown in Table 4. The results reveal that 54.9% of individuals were already living below the national poverty line before making any health care payments. After accounting for OOP payments, the poverty head count increased by 2.7 percentage points. This represents a substantial rise in the poverty estimates, amounting to 5% of the population or 1.98 million individuals. The average deficit to reach the poverty line in the population (i.e. the poverty gap) was KES 3938 before accounting for OOP payments. After accounting for OOP payments, the average deficit increased to KES 4952.

**Table 4:** Poverty head count before and after OOP payments

	**National poverty line**	
	**Gross of health payments**	**Net of health payments**
Poverty headcount	54.9	57.6
Poverty gap	3,937	4952
Normalized poverty gap	26.1	32.8
Normalized mean positive poverty gap	47.5	57.0

## Discussion

The results presented in this paper show great disparities in levels of self-reported illness and health care payment between the poor and the rich. The richest households were more likely to report both outpatient and inpatient illnesses, although wider inequalities were reported for hospital admissions, reflecting the expensive nature of these services. Higher levels of self reported illnesses among the richest population confirm findings reported elsewhere that the poor are more likely to ‘ignore’ illnesses because they cannot afford to seek treatment or to take time off work [27].

Kenyans bear a large burden of OOP payments. Each year, Kenyan households spend close to a tenth of their budget on health care payments. The high levels of OOP payments reflect the health financing system in Kenya, which relies heavily on user fees at the point of service delivery. This financing mechanism does not allow for prepayment, risk pooling and cross-subsidisation. In absolute terms, the richest households spend significantly higher amounts of money on treatment compared to the poorest households. When OOP payments are expressed as a percentage of consumption expenditure, findings reveal a regressive pattern for both outpatient and inpatient illnesses. The poorest households spent five times more of their budget on health care payments compared to the richest population. Poor-rich differences were larger for inpatient compared to outpatient care, indicating that inpatient care is unaffordable to most poor households. High levels of spending among the poor highlight the lack of exemption mechanisms to protect the poor in the Kenyan health sector. The budget share of OOP payments is significantly higher in the urban than in the rural areas, reflecting both differences in socio-economic status and treatment seeking patters. Urban areas have more private providers and larger public health facilities, whose charges are significantly higher than those locate in rural areas. These findings confirm that OOP payments in low-income countries are very regressive as documented in other settings [4,28,29], and highlight the urgent need to protect the poor from high costs of illness.

About 16% and 5% of households incurred health expenditure that exceeded 10% and 40% of total household budget respectively. A larger proportion of households incurred catastrophic payments due to outpatient services compared to inpatient care. About 11% of households spent over 10% of their budget on outpatient treatment, compared to 4.2% for inpatient care. The incidence of catastrophic expenditure at corresponding thresholds is much higher when OOP payments are expressed as a proportion of non-food budget. This increase reflects the greater share of resources spent of food items in Kenya, which is typical of spending patterns in low-income countries. For total OOP payments, 5.6% of households reported payments greater than 40% of total expenditure; this proportion doubled, when the threshold was set relative to share of non-food expenditure. Xu et al. estimated catastrophic spending among Kenyan households using data from a similar survey conducted in 2003 [30]. They found that overall 4.1 per cent of households faced catastrophic health expenditure. About 5.8% and 6.1% of households incurred health care costs over 40% of non-food budget for outpatient and inpatient services respectively. While it is not always possible to directly compare findings due to methodological differences, results presented in this paper suggest that the burden of OOP payments for inpatient care might be decreasing, while that of outpatient care is on the increase. This downward trend in the proportion of households facing catastrophic costs due to inpatient care should be interpreted with caution. It is known that inpatient care is much more expensive than outpatient and it is possible that households might have failed to seek admission due to affordability barriers (particularly the poor). Also, there is a tendency to overestimate annual spending on OOP payments when health costs are scaled to annual estimates. The timing of household surveys also has important implications for levels of self reported illness, treatment seeking patterns and cost burdens [5,27].

Kenya has a mandatory national hospital insurance fund (NHIF) for those working in the formal sector. Informal sector workers can join the NHIF on a voluntary basis. NHIF only caters for costs associated with inpatient care and members have to pay for outpatient services through OOP payments. These findings show that that outpatient care can be expensive and highlight the need to include outpatient benefit packages in the NHIF and other existing prepayment arrangements like community based health insurance. Importantly they highlight the urgent need for Kenya to move towards progressive financing mechanisms that offer financial risk protection for the poor.

The poverty head count accounting for OOP payments was 54.9%. These findings compare closely with those by the World Bank and the Kenya National Bureau of statistics [19,31]. About half 1.48 million Kenyans are pushed below the national poverty line due to OOP payments. The increase in the poverty gap following the change in the poverty line was not only due to individuals falling below the poverty line, but also due to poor individuals falling further below the poverty line once health care payments were subtracted from total consumption expenditure. This shows that OOP payments are a major barrier of development since push non-poor households and trap those who are already poor in it. The role of OOP payments in household poverty has been recognized by many authors [4,15,32]. In Asia, for example, the poverty head count increased by 14% after accounting for health care payments. An additional 2.7% of the population had income that was less than the international poverty line of US$ 1 per day after they paid for health care [26]. Increases were highest in countries that rely heavily on OOP payments as the source of health care funding. These levels are relatively high compared to those reported in this study since the majority of study households were already living in poverty before incurring health care payments. Caution should be taken when interpreting these results for policy. For example, providing financial risk protection for all Kenyans would contribute towards poverty reduction. However, it is unlikely that the impact of these policies will be proportionate to the proportion of individuals impoverished by health care costs as estimated in this paper.

### Limitations

The findings of this study should be interpreted in the context of some limitations. First, data presented are from a cross-sectional household survey. Longitudinal approaches are most suited to capture impacts OOP payments on household living standards. It is however difficult to study a large number of households longitudinally and national representative data that are available are only from cross-sectional surveys. Secondly, the approach assumes that costs are spread evenly over a year. Households might experience peak costs in one month which might have significant implications on their budgets [14]. Thirdly, it has been shown that lost earnings are sometimes more catastrophic than actual payment [6]. Fourthly, estimates of catastrophic health expenditure do not capture individuals who do not seek care due to various barriers. Failing to capture the poorest of the poor could lead to underestimation of the incidence and intensity of catastrophic payments and impoverishment. Finally, poverty levels in Kenya differ between rural and urban areas. It is possible that the majority of households pushed and trapped into poverty due to OOP payments are mainly from rural areas. Regardless of these limitations, important policy lessons can be drawn from the findings presented in this paper.

## Conclusions

It is very clear that the burden of OOP payments is high, Kenyans are becoming poor and many more are being trapped into poverty due to health care payments. The Kenyan government should urgently consider alternative health financing mechanisms that offer financial risk protection to the population. Such approaches, as clearly stated in the WHO 2010 report should encourage risk pooling and income cross-subsidization [33]. Discussions on how best to offer financial risk protection to Kenyans have ongoing for close to a decade now. The results presented in this paper show the urgent need for Kenya to move beyond discussions and implement reforms that will protect the population from health care related impoverishment.

## Competing interests

The authors declare that they have no competing interests.

## Authors’ contribution

JC was responsible for the overall design of the study. JC and TM were involved in data analysis and writing. Both authors read and approved the manuscript.

## Pre-publication history

The pre-publication history for this paper can be accessed here:

http://www.biomedcentral.com/1472-6963/12/413/prepub
